# Supplementary material for: De novo assembly and characterization of the Chinese three-keeled pond turtle (Mauremys reevesii) transcriptome: presence of longevity-related genes
Source: PeerJ. 2016 May 24;4:e2062. doi: 10.7717/peerj.2062 (PMC4888314; doi:10.7717/peerj.2062)
Supplement: File S1 [file peerj-04-2062-s001.docx]

# Repetitive elements and microsatellites analysis of *M. reevesii*

**Transcriptome**

All simple sequence repeats in assembled transcriptome of the turtle were identified using MISA (<http://pgrc.ipk-gatersleben.de/misa/>) with default parameters. All mono-, di-, tri-, tetra-, penta- and hexa-nucleotide motifs were indentified.

Repetitive elements in the pond turtle transcriptome was assessed using RepeatMasker (version open-4.0.5) ([Tarailo‐Graovac & Chen 2009](#_ENREF_2)) with rmblastn (version 2.2.27+) applying the default mode. The underlying database was RepBase (Update 20140131) ([Jurka et al. 2005](#_ENREF_1)) and RM database (version 20140131). The query species was assumed to be vertebrata metazoa.

A total of 101087 SSR (simple sequence repeats) were identified from 75723 transcripts, including mono-, di-, tri-, tetra-, penta-, and hexa-nucleotide repeats. A large amount of transcripts (16.5%) contained repetitive elements and 1.3 repetitive elements per transcript on average (table 1).

A total of 16,147,780 bp (10.62 %) bases were masked with repetitive elements (Table 2). The most abundant type of repetitive elements in the transcriptome was retroelements (6.85%), including LINEs (3.05%), LTR elements (2.33%) and SINEs (1.48%), followed by DNA transposons (3.26%), mostly Tourist/Harbinger (1.22%) and hobo-Activator (1.04%). Additionally, low content of small RNA, satellites, simple repeats and low complexity elements were identified, representing 0.5% of the total sequence length.

Table 1. Statistics of microsatellites identified from pond turtle transcriptome.

| Number of SSR containing sequences | 75723 |
| --- | --- |
| Sequences containing more than 1 SSR | 18019 |
| SSRs present in compound formation | 4240 |
| Identified SSRs | 101087 |
| Mono-nucleotide repeat | 61685 |
| Di-nucleotide repeats | 23880 |
| Tri-nucleotide repeats | 14158 |
| Tetra-nucleotide repeats | 1198 |
| Penta-nucleotide repeats | 101 |
| Hexa-nucleotide repeats | 65 |

**Table 2.** Statistics of repetitive element identified from pond turtle transcriptome.

|  | Number of elements* | Length occupied | Percentage of sequence |
| --- | --- | --- | --- |
| Retroelements | 59455 | 10412100 | 6.85% |
| SINEs: | 16271 | 2241291 | 1.48% |
| Penelope | 3697 | 353442 | 0.23% |
| LINEs: | 29197 | 4633936 | 3.05% |
| CRE/SLACS | 0 | 0 | 0.00% |
| L2/CR1/Rex | 23201 | 3753151 | 2.47% |
| R1/LOA/Jockey | 399 | 95267 | 0.06% |
| R2/R4/NeSL | 89 | 31473 | 0.02% |
| RTE/Bov-B | 828 | 225212 | 0.15% |
| L1/CIN4 | 466 | 76156 | 0.05% |
| LTR elements: | 13967 | 3536873 | 2.33% |
| BEL/Pao | 59 | 3411 | 0.00% |
| Ty1/Copia | 21 | 1051 | 0.00% |
| Gypsy/DIRS1 | 10434 | 2847907 | 1.87% |
| Retroviral | 1970 | 394941 | 0.26% |
|  |  |  |  |
| DNA transposons | 38620 | 4952688 | 3.26% |
| hobo-Activator | 14100 | 1582786 | 1.04% |
| Tc1-IS630-Pogo | 1800 | 181151 | 0.12% |
| En-Spm | 0 | 0 | 0.00% |
| MuDR-IS905 | 0 | 0 | 0.00% |
| PiggyBac | 15 | 833 | 0.00% |
| Tourist/Harbinger | 15405 | 1850400 | 1.22% |
| Other (Mirage, P-element, Transib) | 0 | 0 | 0.00% |
|  |  |  |  |
| Rolling-circles | 0 | 0 | 0.00% |
|  |  |  |  |
| Unclassified: | 3835 | 558916 | 0.37% |
|  |  |  |  |
| Total interspersed repeats: |  | 15984791 | 10.52% |
|  |  |  |  |
| Small RNA: | 3565 | 619975 | 0.41% |
|  |  |  |  |
| Satellites: | 580 | 104591 | 0.07% |
| Simple repeats: | 248 | 38488 | 0.03% |
| Low complexity: | 6 | 914 | 0.00% |
|  |  |  |  |

* Most repeats fragmented by insertions or deletions have been counted as one element

Jurka J, Kapitonov VV, Pavlicek A, Klonowski P, Kohany O, and Walichiewicz J. 2005. Repbase Update, a database of eukaryotic repetitive elements. *Cytogenetic and genome research* 110:462-467.

Tarailo‐Graovac M, and Chen N. 2009. Using RepeatMasker to identify repetitive elements in genomic sequences. *Current Protocols in Bioinformatics*:4.10. 11-14.10. 14.
