# Supplementary material for: De novo assembly and characterization of the Chinese three-keeled pond turtle (Mauremys reevesii) transcriptome: presence of longevity-related genes
Source: PeerJ. 2016 May 24;4:e2062. doi: 10.7717/peerj.2062 (PMC4888314; doi:10.7717/peerj.2062)
Supplement: File S3 [file peerj-04-2062-s003.docx]

| Fragment | Length (bp) | FPKM |
| --- | --- | --- |
| TERT | 3817 | 10.62 |
| TEP1 | 4692 | 10.69 |
| DNA pol β | 1008 | 24.83 |
| DNA pol α catalytic subunit | 2679 | 13.34 |
| DNA pol δ subunit 3 | 1545 | 32.31 |
| DNA pol δ subunit 4 | 336 | 24.6 |
| TRF1 | 405 | 11.72 |
| TRF2 | 555 | 14.02 |
| TPP1 | 1869 | 18.49 |
| RAP1 | 432 | 17.13 |
| POT1 | 2329 | 36.01 |
| TIN2 | 977 | 25.86 |
