# Supplementary figures and images for: De novo assembly and characterization of the Chinese three-keeled pond turtle (Mauremys reevesii) transcriptome: presence of longevity-related genes
Source: PeerJ. 2016 May 24;4:e2062. doi: 10.7717/peerj.2062 (PMC4888314; doi:10.7717/peerj.2062)

Top-Hit species distribution

Species

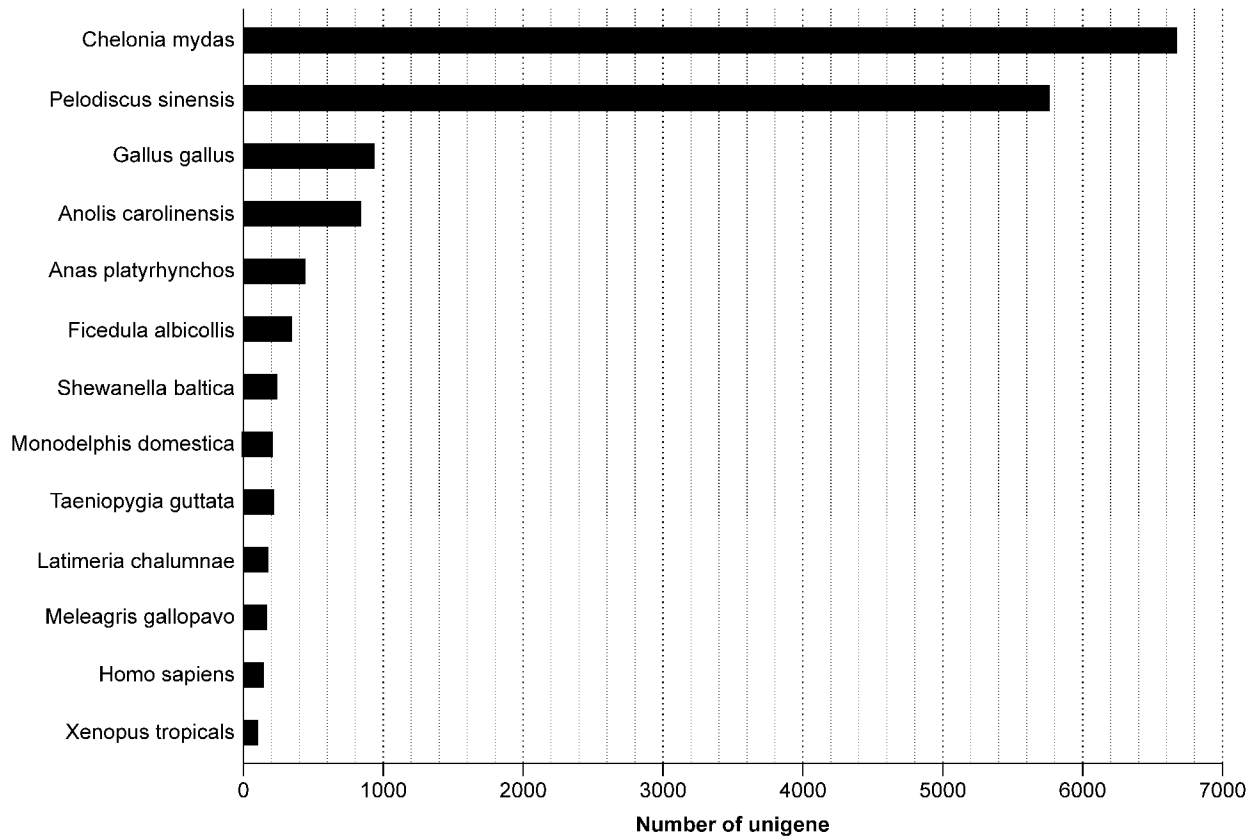

Supplement: Figure S1 [file peerj-04-2062-s004.pdf]
